# Supplementary material for: Callus Culture of Thai Basil Is an Effective Biological System for the Production of Antioxidants
Source: Molecules. 2020 Oct 21;25(20):4859. doi: 10.3390/molecules25204859 (PMC7588007; doi:10.3390/molecules25204859)
Supplement: Supplementary file 1 [file molecules-25-04859-s001.pdf]

Supplementary Materials

Callus Culture of *Ocimum basilicum* L. cv 'Thai Basil' is an Effective Biological System for the Production of Antioxidants Compared to Leaves

Saher Nazir[1], Hasnain Jan[1], Duangjai Tungmunnithum[2,3], Samantha Drouet[2], Muhammad Zia[1], Christophe Hano[3]*, Bilal Haider Abbasi[1]*

[1] Department of Biotechnology, Quaid-i-Azam University, Islamabad, 45320, Pakistan; saher_nazir158@yahoo.com (S.N.); rhasnain849@gmail.com (H.J.); ziachaudhary@gmail.com (M.Z.); bhabbasi@qau.edu.pk (B.H.A.)

[2] Laboratoire de Biologie des Ligneux et des Grandes Cultures, INRAE USC1328, University of Orleans, 45067 Orléans CEDEX 2, France; samantha.drouet@univ-orleans.fr (S.D.); hano@univ-orleans.fr (C.H.)

[3] Department of Pharmaceutical Botany, Faculty of Pharmacy, Mahidol University, Bangkok 10400, Thailand; duangjai.tun@mahidol.ac.th (D.T.)

* Correspondence: bhabbasi@qau.edu.pk (B.H.A.); hano@univ-orleans.fr (C.H.); Tel.: +33-77-698-41-48 (B.H.A.); +33-237-309-753 (C.H.)

Table S1: Callogenesis, morphology and induction frequency of *O. basilicum* cv Thai basil callus culture under various PGRs.

Table S2: Actual values for PCC (Pearson Correlation Coefficient) showing the relation between the different phytochemicals (TPC: total phenolics content; chicoric acid, rosmarinic acid and caffeic acid) in extracts from Thai basil callus cultures and the different antioxidant assays (*in vitro* cell-free: DPPH, ABTS, FRAP and *in vivo*: CAA (cellular antioxidant assay)).

Table S1. Callogenesis, morphology and induction frequency of *O. basilicum* cv Thai basil callus culture under various PGRs.

#	PGRs (mg/L)	Callus initiation (day)	Callus color	Callus texture	Callus induction frequency (%)
**0**	Control (MS)	-	-	-	-
**1**	0.25 GA_3	12	FG	C	28
**2**	0.5 GA_3	11	FG	C	31
**3**	1 GA_3	10	FG	C	40
**4**	2 GA_3	10	FG	C	52
**5**	5 GA_3	12	B	C	30
**6**	10 GA_3	12	B	C	25
**7**	0.25 GA_3+NAA	8	FG	C	75
**8**	0.5 GA_3+NAA	8	FG	C	73
**9**	1 GA_3+NAA	8	FG	C	72
**10**	2 GA_3+NAA	9	SG	C	71
**11**	5 GA_3+NAA	8	SG	C	72
**12**	10 GA_3+NAA	8	SG	C	72
**13**	0.25 BAP	9	LG	C	58
**14**	0.5 BAP	9	LG	C	60
**15**	1 BAP	9	LG	C	62
**16**	2 BAP	9	LG	C	70
**17**	5 BAP	9	YG	C	20
**18**	10 BAP	10	YG	C	30
**19**	0.25 BAP +NAA	8	SG	C	80
**20**	0.5 BAP + NAA	8	SG	C	86
**21**	1 BAP +NAA	7	SG	C	90
**22**	2 BAP +NAA	7	SG	C	92
**23**	**5 BAP +NAA**	**7**	**SG**	**C**	**95**
**24**	10 BAP +NAA	8	SG	C	90

C compact, LG light green, FG fresh green, B brown, SG snowy green, YG yellowish green

Table S2: Actual values for PCC (Pearson Correlation Coefficient) showing the relation between the different phytochemicals (TPC: total phenolics content; chicoric acid, rosmarinic acid and caffeic acid) in extracts from Thai basil callus cultures and the different antioxidant assays (*in vitro* cell-free: DPPH, ABTS, FRAP and *in vivo*: CAA (cellular antioxidant assay)).

	TPC	Chicoric acid	Rosmarinic acid	Caffeic acid
Biomass	0.780 ***	0.469 *	0.675 ***	0.259
DPPH	0.172	0.238 *	0.253 *	0.419 *
ABTS	0.675 ***	0.709 ***	0.808 ***	0.551
FRAP	0.081	-0.137 *	-0.193 *	-0.510 *
CAA	0.142	0.187 ***	0.399 **	0.849 **

*** significant $p < 0.001$; ** significant $p < 0.01$; * significant $p < 0.05$.
